# Four-body atomic potential for modeling protein-ligand binding affinity: application to enzyme-inhibitor binding energy prediction

**DOI:** 10.1186/1472-6807-13-S1-S1

**Published:** 2013-11-08

**Authors:** Majid Masso

**Affiliations:** 1 Laboratory for Structural Bioinformatics, School of Systems Biology, George Mason University, 10900 University Blvd. MS 5B3, Manassas, Virginia 20110, USA

## Abstract

**Background:**

Models that are capable of reliably predicting binding affinities for protein-ligand complexes play an important role the field of structure-guided drug design.

**Methods:**

Here, we begin by applying the computational geometry technique of Delaunay tessellation to each set of atomic coordinates for over 1400 diverse macromolecular structures, for the purpose of deriving a four-body statistical potential that serves as a topological scoring function. Next, we identify a second, independent set of three hundred protein-ligand complexes, having both high-resolution structures and known dissociation constants. Two-thirds of these complexes are randomly selected to train a predictive model of binding affinity as follows: two tessellations are generated in each case, one for the entire complex and another strictly for the isolated protein without its bound ligand, and a topological score is computed for each tessellation with the four-body potential. Predicted protein-ligand binding affinity is then based on an empirically derived linear function of the difference between both topological scores, one that appropriately scales the value of this difference.

**Results:**

A comparison between experimental and calculated binding affinity values over the two hundred complexes reveals a Pearson's correlation coefficient of *r *= 0.79 with a standard error of *SE *= 1.98 kcal/mol. To validate the method, we similarly generated two tessellations for each of the remaining protein-ligand complexes, computed their topological scores and the difference between the two scores for each complex, and applied the previously derived linear transformation of this topological score difference to predict binding affinities. For these one hundred complexes, we again observe a correlation of *r *= 0.79 (*SE *= 1.93 kcal/mol) between known and calculated binding affinities. Applying our model to an independent test set of high-resolution structures for three hundred diverse enzyme-inhibitor complexes, each with an experimentally known inhibition constant, also yields a correlation of *r *= 0.79 (*SE *= 2.39 kcal/mol) between experimental and calculated binding energies.

**Conclusions:**

Lastly, we generate predictions with our model on a diverse test set of one hundred protein-ligand complexes previously used to benchmark 15 related methods, and our correlation of *r *= 0.66 between the calculated and experimental binding energies for this dataset exceeds those of the other approaches. Compared with these related prediction methods, our approach stands out based on salient features that include the reliability of our model, combined with the rapidity of the generated predictions, which are less than one second for an average sized complex.

## Background

**Figure 1:**
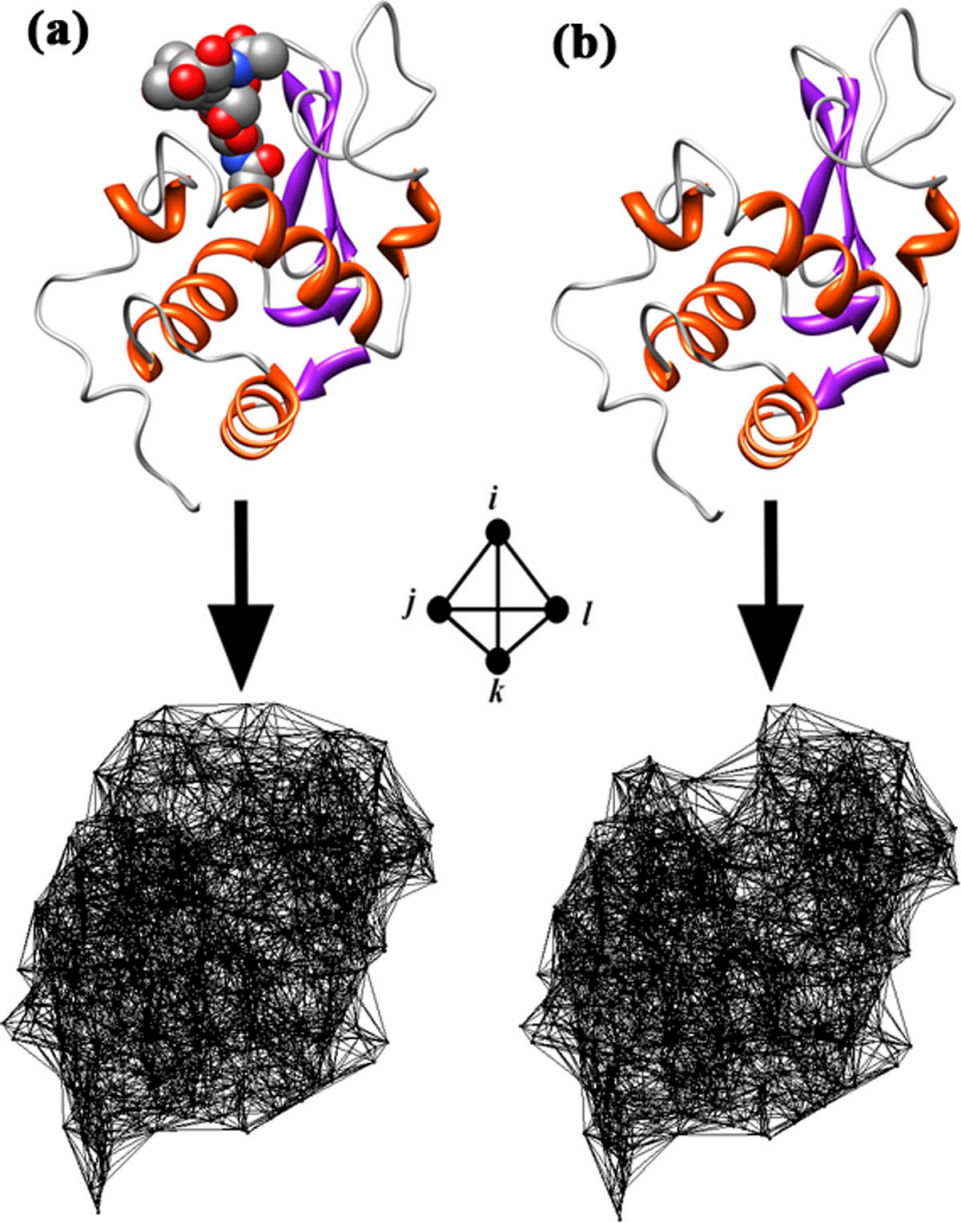
**Atomic Delaunay tessellation of the hen egg-white lysozyme (a) in complex with bound ligand NAG (N-Acetyl-D-Glucosamine) and (b) without the bound ligand (PDB accession code: **1HEW**)**.

Experimental high-throughput screening processes that drive structure-guided drug design efforts are effective tools for the identification of candidate molecular ligands that may tightly bind a target protein; however, such an approach often proves to be a costly endeavor, in terms of both time and financial expense, one that can potentially be alleviated with reliable *in silico *protein-ligand binding affinity models to assist in winnowing the search space [1]. A diverse array of computational approaches to model binding affinity have been described in the literature, each of which focuses on a unique combination of physicochemical properties and interactions: X-Score [2], Lig-Score [3], DrugScore [4], SFCscore [5], AutoDock4 [6], ITScore [7,8], and PHOENIX [9] are just a few examples of such predictive tools. Here we describe our development of a model for predicting protein-ligand binding energy that relies on Delaunay tessellation, a computational geometry technique [10], for the purpose of objectively capturing nearest neighbor atomic four-body interactions in the structures of macromolecular complexes (Figure 1).

First, we compute the propensities for occurrence of all atomic quadruplet interactions by applying the tessellation procedure to atomic coordinates for a diverse cross-section of over 1400 high-resolution macromolecular crystal structures, and the data collected are used in generating an atomic four-body potential. Tasked with distinguishing native structures as having global energy minima relative to decoys, our knowledge-based potential performs well compared to several related atomic energy functions [11,12]; however this work constitutes substantial research outside the immediate focus of this study, and accordingly it will be reported elsewhere. Next, we apply our atomic potential to a separate dataset of three hundred diverse protein-ligand complexes, each selected for having both a solved high-resolution crystal structure and a known dissociation constant (*k_d_*), the latter quantity being useful for determining the Gibbs free energy of binding (Δ*G*). Two thirds of the complexes are randomly selected to train our predictive model of binding affinity: in each case, the entire complex is tessellated and then scored using the four-body potential, as is the structure of the isolated protein without its bound ligand, and we derive an empirical linear function of the difference between these scores to predict Δ*G *values. The remaining one hundred complexes are then used to validate the capability of the trained linear model to predict binding energies for new protein-ligand complexes.

The steps taken to develop our model formed the basis of a recently published companion study [13], and here we begin by carefully outlining those details below, since they lay the foundation for the next stage of the work to be presented. In particular, the model is subsequently applied to the prediction of binding affinities for an independent, diverse test set of three hundred enzyme-inhibitor complexes for which high-resolution crystal structures, as well as experimentally determined inhibition constants (*k_i_*), are available. Also, model performance is comprehensively benchmarked against a number of related methods from the literature.

## Methods

### Datasets

High-resolution (≤ 2.2Å) crystallographic structures for 1417 macromolecular complexes (Additional file 1), culled using the PISCES server [14] and having protein chains that share low (< 30%) sequence identity, were selected to develop the four-body statistical potential. Dataset diversity is also reflected in the fact that the complexes consist of both single chain and multimeric proteins, many of which have bound ligands in the form of either small molecules or peptides. Each complex has a coordinate file deposited in the Protein Data Bank (PDB) [15], and following the removal of all hydrogen atoms and water molecules, Delaunay tessellation is applied to each structure file by using all the remaining atomic coordinates.

In order to train and validate our model for predicting binding affinity, we selected another diverse set of three hundred protein-ligand complexes (Additional file 2) from the Binding MOAD [16,17] database. The Binding MOAD is a repository for all protein-ligand complexes that have high-resolution (≤ 2.5Å) structures deposited in the PDB, and if available, published experimental binding energy data. Focusing specifically on a non-redundant subset of the Binding MOAD, both to ensure diversity of complexes as well as to minimize bias due to over-represented structures, we identified three hundred complexes having both PDB coordinate files as well as experimental dissociation constants (*k_d_*). The PDB accession codes and *k_d _*values for the protein-ligand complexes are tabulated in Additional file 2, as is the identity of the subset (200 for training, and 100 for validation) into which each is randomly placed.

### Software and performance measurements

We use the Qhull software package [18] to carry out the atomic Delaunay tessellations, Matlab (Version 7.0.1.24704 (R14) Service Pack 1) to produce graphical depictions of the tessellations, and the UCSF Chimera software package [19] to generate all other molecular visualizations in this study. Codes to perform all data formatting and analyses tasks are written in the Perl programming language.

Given the dissociation constant (*k_d_*) for a protein-ligand complex, the standard Gibbs free energy of binding (Δ*G*, in units of kcal/mol) can be determined using

(1) $$\text{Δ}G=RT\text{ ln}\left(k_{d}\right)=0.592\times \text{ln}\left(k_{d}\right),$$

where *R *= 1.986 ×10^-3 ^kcal K^-1 ^mol^-1 ^is the gas constant and *T *= 298° K is the absolute temperature. We evaluate the agreement between known (*x_i_*) and predicted (*y_i_*) binding energies by reporting the Pearson's correlation coefficient

(2) $$r=\frac{\sum \left(x_{i}-\bar{x}\right)\left(y_{i}-\bar{y}\right)}{\sqrt{\left[\sum {\left(x_{i}-\bar{x}\right)}^{2}\right]\;\left[\sum {\left(y_{i}-\bar{y}\right)}^{2}\right]}},$$

the standard error of the predictions

(3) $$\begin{array}{cc} SE & =\sqrt{\frac{\left(1-r^{2}\right)\sum {\left(y_{i}-\bar{y}\right)}^{2}}{n-2}}\\ & =\sqrt{\left[\frac{1}{n\left(n-2\right)}\right]\;\left[n\sum {y_{i}}^{2}-{\left(\sum y_{i}\right)}^{2}-\frac{{\left[n\sum x_{i}y_{i}-\left(\sum x_{i}\right)\left(\sum y_{i}\right)\right]}^{2}}{n\sum {x_{i}}^{2}-{\left(\sum x_{i}\right)}^{2}}\right]} \end{array},$$

and the equation of the fitted regression line.

## Results

### Four-body statistical potential

To generate our knowledge-based potential, a six-letter alphabet (C, N, O, S, M = all metals, X = all other non-metals) is used for labelling all atoms (excluding hydrogens and water molecules). The Qhull software uses the 3-dimensional (3D) coordinates of atoms in a PDB file to generate a Delaunay tessellation of the structure, a space-filling convex hull formed by hundreds of solid, non-overlapping, irregular tetrahedra whose vertices are the 3D atomic points. Each atom serves as a vertex, with most being shared by numerous adjacent tetrahedra, and every tetrahedral simplex objectively identifies a quadruplet of nearest neighbor atoms at its four vertices. To ensure this is indeed the case, we eliminate all edges longer than 8Å immediately upon tessellation, which is in agreement with related research in this arena at the atomic [20] and residue [21,22] levels of coarse-graining. The combined total number of tetrahedra remaining for analysis after tessellating the 1417 PDB coordinate files is provided in Table 1, as are the total number of atoms of each type as well as their relative frequencies.

**Table 1:** Summary data for the 1417 PDB structure files.

Atom Types	Count	Proportion
C	3,612,988	0.633193
N	969,253	0.169866
O	1,088,410	0.190749
S	28,502	0.004995
(all metals) M	2,529	0.000443
(all other non-metals) X	4,299	0.000754
		
Total atom count:	5,705,981	
		
Total tetrahedron count:	34,504,737	

Without regards to the ordering of a quadruplet of atoms (i.e, all permutations of the four letters are non-unique and represent the same quadruplet), and allowing for the repeated occurrence of atom types in any given quadruplet (i.e., letters may appear more than once in a quadruplet), there are 126 possible types of atomic quadruplets that can be enumerated based on the use of a 6-letter atomic alphabet (Table 2). For each quadruplet (*i*,*j*,*k*,*l*), we define *f_ijkl _*as the observed proportion of all tetrahedral simplices obtained by tessellating all 1417 structures to have those four types of atoms at the vertices; similarly, we let *p_ijkl _*represent the rate expected by chance, which is based on relative frequencies of the six atom types in the structures (Table 1) and calculated using a multinomial background distribution given by

**Table 2:** Atomic four-body statistical potential.

Quad	Count	*f_ijkl_*	*p_ijkl_*	*s_ijkl_*	Quad	Count	*f_ijkl_*	*p_ijkl_*	*s_ijkl_*
CCCC	4015872	0.116386	0.160748	-0.140244	MMNS	363	1.05E-05	2.00E-09	3.720958
CCCM	1592	4.61E-05	0.000450	-0.989223	MMNX	0	0	3.02E-10	--
CCCN	4025206	0.116657	0.172495	-0.169866	MMOO	306	8.87E-06	4.29E-08	2.315530
CCCO	6202159	0.179748	0.193701	-0.032467	MMOS	104	3.01E-06	2.25E-09	3.127729
CCCS	293157	0.008496	0.005072	0.224008	MMOX	3	8.69E-08	3.39E-10	2.409325
CCCX	2796	8.10E-05	0.000765	-0.975047	MMSS	254	7.36E-06	2.94E-11	5.398477
CCMM	132	3.83E-06	4.73E-07	0.908235	MMSX	2	5.80E-08	8.87E-12	3.815151
CCMN	3318	9.62E-05	0.000362	-0.575981	MMXX	0	0	6.69E-13	--
CCMO	5325	0.000154	0.000407	-0.420893	MNNN	1030	2.99E-05	8.69E-06	0.535960
CCMS	2293	6.65E-05	1.07E-05	0.795108	MNNO	1128	3.27E-05	2.93E-05	0.047955
CCMX	15	4.35E-07	1.61E-06	-0.567697	MNNS	561	1.63E-05	7.67E-07	1.326526
CCNN	1797552	0.052096	0.069412	-0.124635	MNNX	5	1.45E-07	1.16E-07	0.098041
CCNO	8233136	0.238609	0.155892	0.184864	MNOO	3744	0.000109	3.29E-05	0.518626
CCNS	124653	0.003613	0.004082	-0.053081	MNOS	314	9.10E-06	1.72E-06	0.723107
CCNX	2007	5.82E-05	0.000616	-1.024729	MNOX	29	8.40E-07	2.60E-07	0.510083
CCOO	3366568	0.097568	0.087528	0.047161	MNSS	793	2.30E-05	2.25E-08	3.008398
CCOS	198630	0.005757	0.004584	0.098905	MNSX	5	1.45E-07	6.80E-09	1.328573
CCOX	4626	0.000134	0.000691	-0.712426	MNXX	9	2.61E-07	5.13E-10	2.706383
CCSS	15288	0.000443	6.00E-05	0.868158	MOOO	5430	0.000157	1.23E-05	1.106856
CCSX	144	4.17E-06	1.81E-05	-0.637352	MOOS	156	4.52E-06	9.67E-07	0.669977
CCXX	143	4.14E-06	1.37E-06	0.482159	MOOX	168	4.87E-06	1.46E-07	1.523669
CMMM	23	6.67E-07	2.21E-10	3.480397	MOSS	210	6.09E-06	2.53E-08	2.380989
CMMN	144	4.17E-06	2.54E-07	1.216422	MOSX	4	1.16E-07	7.64E-09	1.181307
CMMO	256	7.42E-06	2.85E-07	1.415945	MOXX	55	1.59E-06	5.76E-10	3.442148
CMMS	662	1.92E-05	7.46E-09	3.410480	MSSS	62	1.80E-06	2.21E-10	3.910199
CMMX	1	2.90E-08	1.12E-09	1.411130	MSSX	2	5.80E-08	1.00E-10	2.763224
CMNN	2474	7.17E-05	9.72E-05	-0.132029	MSXX	0	0	1.51E-11	--
CMNO	6267	0.000182	0.000218	-0.079754	MXXX	16	4.64E-07	7.58E-13	5.786451
CMNS	2588	7.50E-05	5.72E-06	1.118068	NNNN	3878	0.000112	0.000833	-0.869698
CMNX	26	7.54E-07	8.62E-07	-0.058415	NNNO	46665	0.001352	0.003740	-0.441730
CMOO	8481	0.000246	0.000123	0.302308	NNNS	460	1.33E-05	9.79E-05	-0.866046
CMOS	1010	2.93E-05	6.42E-06	0.659069	NNNX	34	9.85E-07	1.48E-05	-1.175817
CMOX	68	1.97E-06	9.68E-07	0.308765	NNOO	340620	0.009872	0.006299	0.195102
CMSS	2047	5.93E-05	8.40E-08	2.848813	NNOS	5637	0.000163	0.000330	-0.305233
CMSX	13	3.77E-07	2.53E-08	1.172117	NNOX	302	8.75E-06	4.98E-05	-0.754766
CMXX	6	1.74E-07	1.91E-09	1.958862	NNSS	311	9.01E-06	4.32E-06	0.319427
CNNN	102035	0.002957	0.012414	-0.623046	NNSX	6	1.74E-07	1.30E-06	-0.874705
CNNO	1995038	0.057819	0.041821	0.140679	NNXX	5	1.45E-07	9.83E-08	0.168652
CNNS	15892	0.000461	0.001095	-0.376176	NOOO	171147	0.004960	0.004716	0.021937
CNNX	578	1.68E-05	0.000165	-0.993919	NOOS	10697	0.000310	0.000370	-0.077374
CNOO	2734639	0.079254	0.046962	0.227273	NOOX	3102	8.99E-05	5.59E-05	0.206513
CNOS	95438	0.002766	0.002460	0.050981	NOSS	922	2.67E-05	9.70E-06	0.440012
CNOX	2168	6.28E-05	0.000371	-0.771173	NOSX	12	3.48E-07	2.93E-06	-0.925060
CNSS	4264	0.000124	3.22E-05	0.584024	NOXX	61	1.77E-06	2.21E-07	0.903627
CNSX	37	1.07E-06	9.71E-06	-0.957113	NSSS	33	9.56E-07	8.47E-08	1.052833
CNXX	61	1.77E-06	7.33E-07	0.382553	NSSX	0	0	3.83E-08	--
COOO	524994	0.015215	0.017579	-0.062707	NSXX	0	0	5.78E-09	--
COOS	34429	0.000998	0.001381	-0.141141	NXXX	3	8.69E-08	2.91E-10	2.475964
COOX	23801	0.000690	0.000208	0.520038	OOOO	34212	0.000992	0.001324	-0.125549
COSS	4380	0.000127	3.62E-05	0.545326	OOOS	4240	0.000123	0.000139	-0.052504
COSX	58	1.68E-06	1.09E-05	-0.812243	OOOX	9553	0.000277	2.09E-05	1.121777
COXX	65	1.88E-06	8.23E-07	0.359781	OOSS	300	8.69E-06	5.45E-06	0.203077
CSSS	285	8.26E-06	3.16E-07	1.417735	OOSX	36	1.04E-06	1.64E-06	-0.197264
CSSX	5	1.45E-07	1.43E-07	0.006247	OOXX	128	3.71E-06	1.24E-07	1.476181
CSXX	4	1.16E-07	2.15E-08	0.730845	OSSS	38	1.10E-06	9.51E-08	1.063748
CXXX	9	2.61E-07	1.08E-09	2.381656	OSSX	3	8.69E-08	4.30E-08	0.305472
MMMM	83	2.41E-06	3.86E-14	7.794725	OSXX	0	0	6.49E-09	--
MMMN	37	1.07E-06	5.92E-11	4.258301	OXXX	2	5.80E-08	3.26E-10	2.249518
MMMO	29	8.40E-07	6.64E-11	4.102142	SSSS	6	1.74E-07	6.23E-10	2.446092
MMMS	379	1.10E-05	1.74E-12	6.800300	SSSX	0	0	3.76E-10	--
MMMX	0	0	2.62E-13	--	SSXX	0	0	8.50E-11	--
MMNN	83	2.41E-06	3.40E-08	1.849597	SXXX	0	0	8.55E-12	--
MMNO	102	2.96E-06	7.64E-08	1.587734	XXXX	0	0	3.22E-13	--

(4) $$p_{ijkl}=\frac{4!}{\overset{6}{\underset{n=1}{\prod }}\left(t_{n}!\right)}\overset{6}{\underset{n=1}{\prod }}a_{n}^{t_{n}}\text{,where }\overset{6}{\underset{n=1}{\sum }}a_{n}=1\text{ and }\overset{6}{\underset{n=1}{\sum }}t_{n}=4.$$

In Eq. (4), *a_n _*is the relative frequency of atom type *n*, while *t_n _*counts how many times atom type *n *appears in the quadruplet (*i*,*j*,*k*,*l*). As a consequence of the inverted Boltzmann principle [23], the score *s_ijkl _*= log (*f_ijkl _*/ *p_ijkl_*) is proportional to the energy of quadruplet atomic interaction, and the set of 126 scored atomic quadruplets defines our four-body statistical potential (Table 2).

### Topological scores

In order to develop our predictive model, the four-body potential is applied to the dataset of three hundred protein-ligand complexes compiled from the Binding MOAD in the following manner. For each complex, the atomic coordinates (excluding hydrogens and water molecules) in the PDB file are tessellated (edges longer than 8Å removed), each tetrahedron in the tessellation is scored using Table 2 according to the four atoms at its vertices, and a normalized topological score (*Q*) is calculated to be the sum of all the tetrahedral scores divided by the number of tetrahedra in the tessellation, a quantity that can be summarized compactly by the equation

(5) $$Q=\frac{1}{N}\underset{\left(i,j,k,l\right)}{\sum }s_{ijkl}$$

Next, atomic coordinates of the ligand are removed from the PDB file of the complex, and the procedure is repeated to compute *Q *for the isolated protein (Figure 1). Lastly, we define the topological score difference

(6) $$\text{Δ}Q=Q_{\text{complex}}-Q_{\text{protein}}$$

for the complex. In the next section, we compare computed Δ*Q *quantities with known Δ*G *values for these complexes in order to develop a model for predicting binding energy. An important underlying assumption in this formulation is that ligand size is small enough so that tetrahedra formed at the interface with the protein dominate purely internal atomic interactions within the ligand. The calculated *Q *values, for structures of the three hundred protein-ligand complexes, as well as the isolated proteins without their bound ligands, are tabulated in Additional file 2.

### Predictive model of binding energy

**Figure 2:**
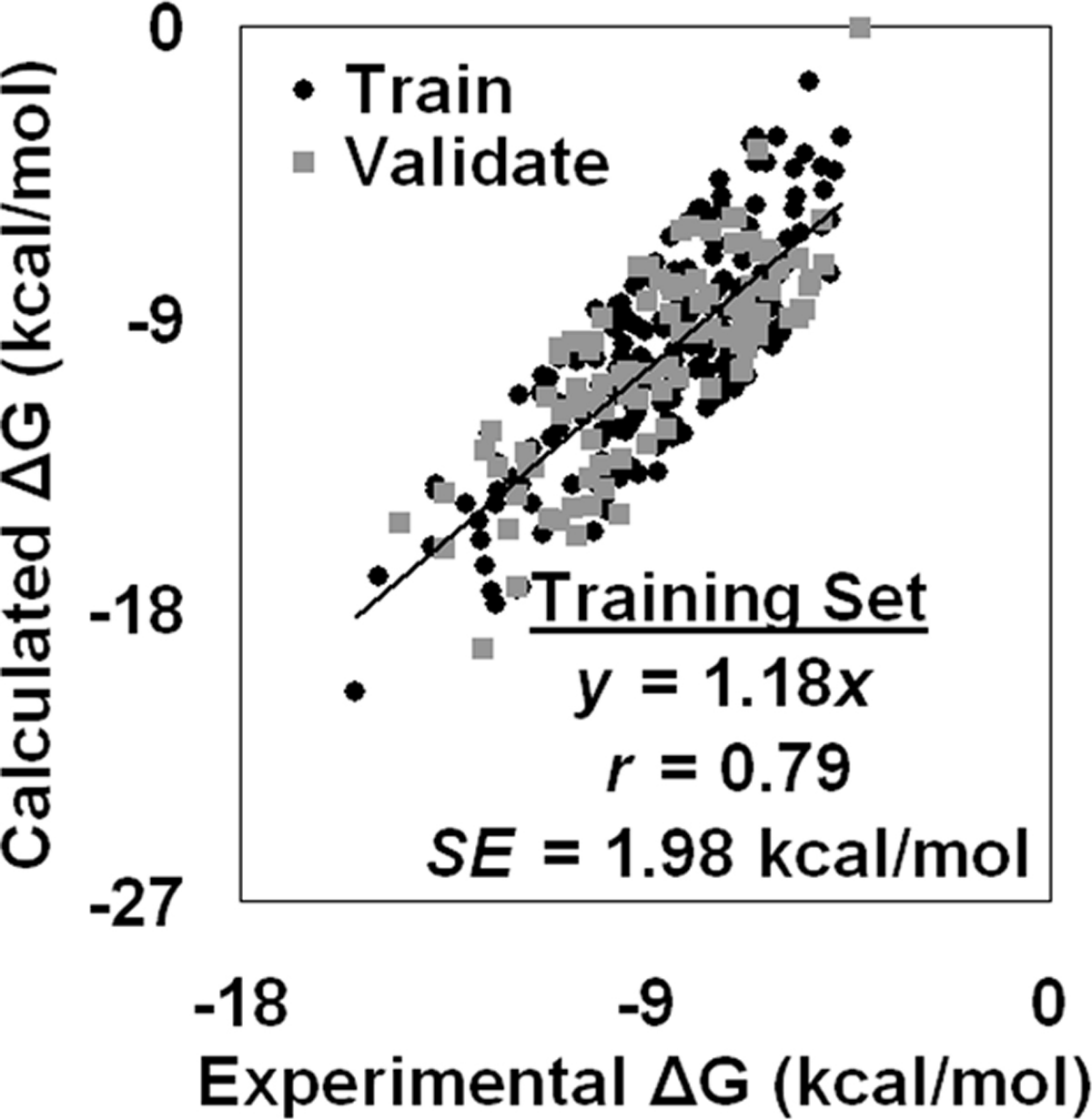
**Scatter plot of calculated versus experimental binding energies for the dataset of three hundred protein-ligand complexes**.

A comparison of the calculated Δ*Q *quantities for our training set of two hundred randomly selected complexes with their experimental Δ*G *values (Δ*G*_exp_) reveals a correlation coefficient of *r *= 0.79. However, the Δ*Q *values are not uniform in sign, and they are on a significantly smaller scale relative to the standard Gibbs free energy of binding (Δ*G*_exp_) data; hence, they cannot be used directly as a representation of predicted Δ*G *values (Δ*G*_calc_). Both issues related to Δ*Q *values for the training data are addressed with an empirically derived linear function

(7) $$\text{Δ}G_{\text{calc}}=\left(1/0.0003\right)\times \text{Δ}Q-10.49,$$

resulting in negative Δ*G*_calc _values in each case that also scale similarly to Δ*G*_exp_. Owing to Δ*G*_calc _arising from a simple linear transformation of the Δ*Q *values, Δ*G*_calc _and Δ*G*_exp _also display a correlation of *r *= 0.79 (*SE *= 1.98 kcal/mol) with a fitted regression line of *y *= 1.18*x *(Figure 2).

Turning next to the validation set of one hundred complexes, we obtain Δ*G*_calc _values from their computed Δ*Q *quantities by utilizing the linear model given in Eq. (7) that we empirically derived from the training data. The predicted Δ*G*_calc _and known Δ*G*_exp _values for these complexes again display a correlation of *r *= 0.79 (*SE *= 1.93 kcal/mol) with a fitted regression line of *y *= 1.11*x *- 0.63, and a scatter plot of the validation data is superimposed over that of the training data in Figure 2. Tabulated in Additional file 2 are Δ*G*_exp _and Δ*G*_calc _values for all three hundred protein-ligand complexes.

## Discussion

### Enzyme-inhibitor binding affinity prediction

In order to test the utility of our model through a practical application, we predict binding affinities for a diverse dataset of three hundred enzyme-inhibitor complexes (Additional file 3), independent of those protein-ligand complexes used for training and validation, which are annotated with their respective experimental inhibition constants (*k_i_*) in the non-redundant Binding MOAD. Analogous to Eq. (1), we obtain the standard Gibbs free energy of binding for each complex with the equation

(8) $$\text{Δ}G_{\text{exp}}=RT\text{ ln}\left(k_{i}\right)=0.592\times \text{ln}\left(k_{i}\right),$$

where *R *= 1.986 ×10^-3 ^kcal K^-1 ^mol^-1 ^is the gas constant and *T *= 298° K is the absolute temperature. Tabulated in Additional file 3 are the PDB accession codes of the high-resolution (≤ 2.5Å) crystallographic structures, as well as the *k_i _*and Δ*G*_exp _values, corresponding to these enzyme-inhibitor complexes.

Next, we use the atomic coordinates (hydrogen atoms and water molecules excluded) provided by the PDB structure file for each complex to generate a Delaunay tessellation (subject to an 8Å edge-length cutoff), from which we obtain a normalized topological score (*Q*_complex_) by employing Eq. (5) in conjunction with our atomic four-body statistical potential (Table 2). In a similar fashion, we generate a normalized topological score for the isolated protein without the bound inhibitor (*Q*_protein_), by tessellating a modified version of the PDB file that excludes the atomic coordinates for the inhibitor. Lastly, we calculate the difference (Δ*Q*) between these normalized topological scores according to Eq. (6), which is subsequently used by our model in Eq. (7) to yield a prediction for the enzyme-inhibitor binding affinity (Δ*G*_calc_). All normalized topological score and calculated binding affinity data are also tabulated in Additional file 3.

**Figure 3:**
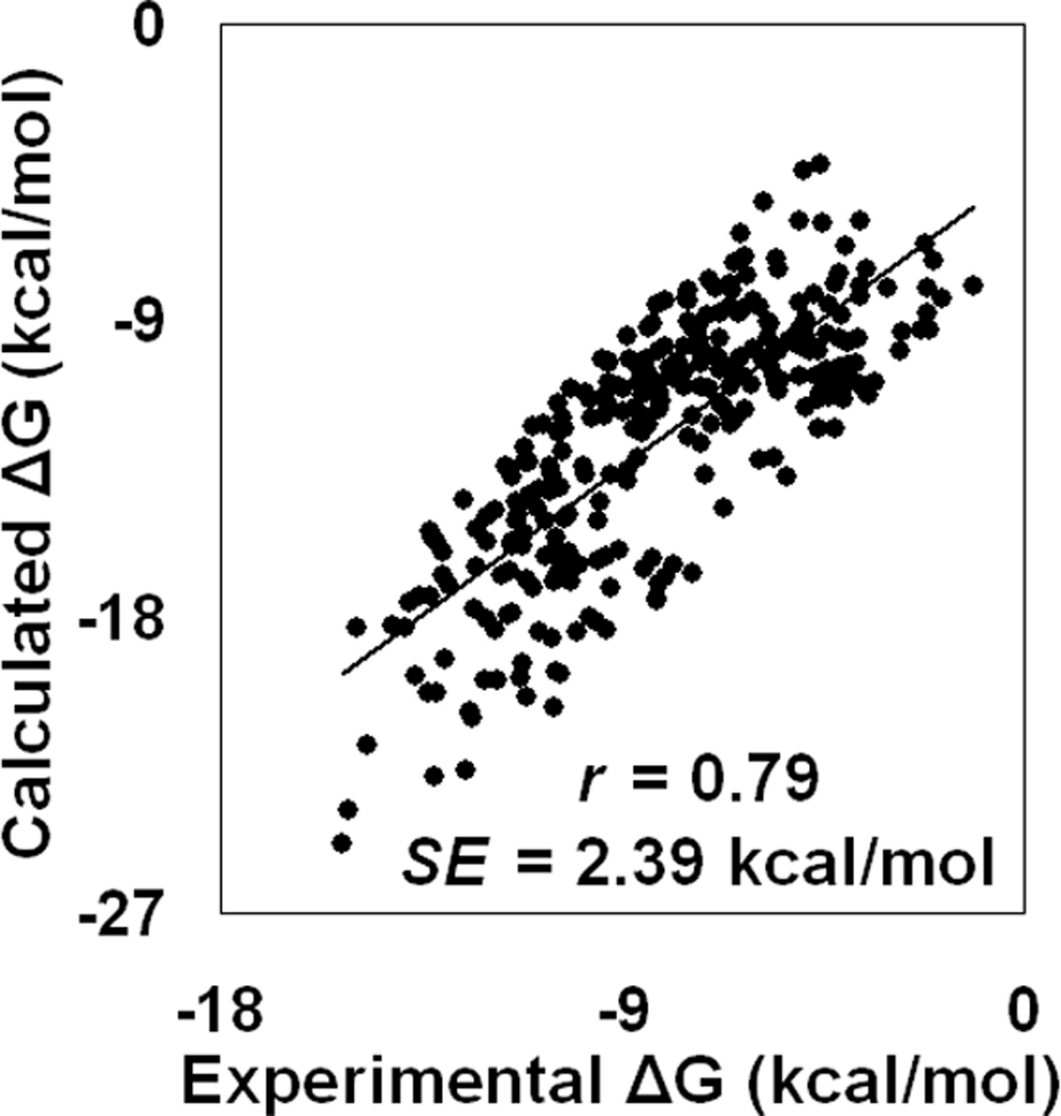
**Scatter plot of calculated versus experimental binding energies for the dataset of three hundred enzyme-inhibitor complexes**.

For this dataset of three hundred enzyme-inhibitor complexes, the calculated Δ*Q *values and the experimental binding affinity Δ*G*_exp _data display a correlation of *r *= 0.79; likewise, as discussed previously, the correlation between Δ*G*_calc _and Δ*G*_exp _is similarly given by *r *= 0.79, in this case with a calculated standard error for the predictions of *SE *= 2.39 kcal/mol (Figure 3).

### Comparisons to related methods

In the same way that our predictive model of protein-ligand binding affinity is evaluated on a test set of three hundred enzyme-inhibitor complexes as described in the previous section, other related methods similarly use test sets of complexes to validate their models. Hence, to directly compare our performance to that of other methods, binding affinity predictions are generated using our approach for complexes that form their test sets. Starting with X-Score, Wang *et al*. [2] report predictions with their model on a test set of ten complexes that reflect a correlation of *r *= 0.67 between experimental and predicted binding affinity (right hand columns of Table 3 in [2], predicted data are in parentheses), with a fitted regression line of *y *= 0.31*x *+ 3.78. On the identical dataset, predictions obtained with our model yield a correlation of *r *= 0.72 and fitted regression line of *y *= 1.26*x *- 1.20, results that signify a clear improvement over those of X-Score (Table 3 of this manuscript, which also reproduces the X-Score data).

**Table 3:** Comparing experimental binding affinity values for 10 protein-ligand complexes with predicted values obtained using both X-Score and the model developed in this study.

	*pk_d_*		Δ*G*	
		
PDB code	Exp.	X-Score	Exp.	Our model
1ABE	6.52	5.25	-8.887610045	-10.3129
1ADB	8.40	8.01	-11.45029515	-23.3388
1ADD	6.74	5.36	-9.187498728	-13.1243
1AF2	3.10	4.90	-4.225704163	-10.0826
1ANF	5.46	6.03	-7.442691848	-9.59131
1CBX	6.35	5.74	-8.655877882	-11.6284
1DBM	9.44	6.65	-12.86795074	-14.9936
1DHF	7.40	5.27	-10.08716478	-13.1753
1GST	4.68	5.21	-6.379450155	-5.48917
1HPV	9.22	6.28	-12.56806206	-16.0903
		
	X-Score: *r *= 0.67		Our model: *r *= 0.72	

Experimental Δ*G *data for the complexes are derived from the experimental *pk_d_* values.

**Figure 4:**
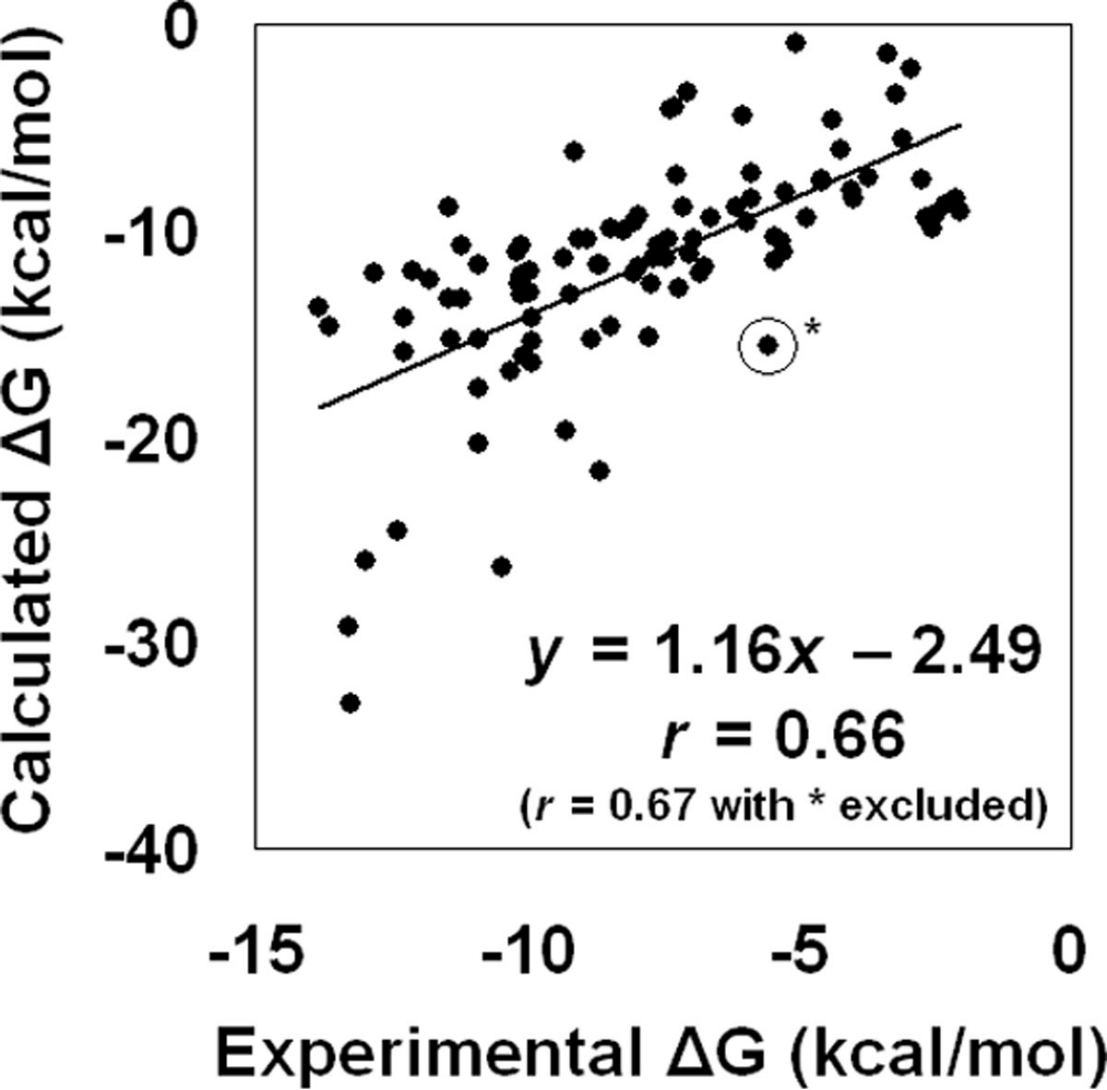
**Scatter plot of calculated versus experimental binding energies using our model for the benchmarking dataset of one hundred enzyme-inhibitor complexes**.

Turning next to ITScore, we discover that Huang *et al*. [8] utilize a benchmarking test set consisting of one hundred protein-ligand complexes, originally constructed by Wang *et al*. [24], to compare their scoring function and 14 other methods by ranking the respective Pearson's correlation coefficients (*r*) between experimental and predicted binding affinities. The test set is diverse, consisting of 43 different proteins as well as binding affinities that span nearly nine orders of magnitude. By generating binding affinity predictions for these one hundred complexes with our model and calculating their correlation with the experimental data, we can subsequently determine our ranking among these 15 related approaches: ITScore [8], X-Score [2], DFIRE [25], DrugScore^CSD ^[26], DrugScore^PDB ^[4], Cerius2/PLP [27,28], SYBYL/G-Score [29], SYBYL/D-Score [30], SYBYL/ChemScore [31], Cerius2/PMF [32], DOCK/FF [30], Cerius2/LUDI [33,34], Cerius2/Lig-Score [35], SYBYL/F-Score [36], and AutoDock [37]. The results of our predictions are summarized in Figure 4, which provides a scatter plot of calculated versus experimental binding energies for this dataset of one hundred complexes. The plot reflects a correlation of *r *= 0.66 (*r *= 0.67 with one outlier complex excluded), surpassing all of the other methods (Table 4, data for the other methods are obtained from Table 3 in [8]) and validating the reliability of our approach.

**Table 4:** Benchmarking correlations between calculated and experimental binding energies using our model and 15 related methods on 100 protein-ligand complexes.

Method	Type	Correlation coefficient (*r*)
**Our model**	**Knowledge-based**	**0.66**
ITScore	Iterative score	0.65
X-Score	Empirical	0.64
DFIRE	Knowledge-based	0.63
DrugScore^CSD^	Knowledge-based	0.62
DrugScore^PDB^	Knowledge-based	0.60
Cerius2/PLP	Empirical	0.56
SYBYL/G-Score	Force-field-based	0.56
SYBYL/D-Score	Force-field-based	0.48
SYBYL/ChemScore	Empirical	0.47
Cerius2/PMF	Knowledge-based	0.40
DOCK/FF	Force field	0.40
Cerius2/LUDI	Empirical	0.36
Cerius2/Lig-Score	Force-field-based	0.35
SYBYL/F-Score	Empirical	0.30
AutoDock	Force-field-based	0.05

## Conclusions

Delaunay tessellation of atomic coordinates in a diverse dataset of macromolecular structures objectively identifies four-body atomic interactions, providing the raw data for developing a knowledge-based atomic four-body statistical contact potential. This potential is used to score a separate diverse set of three hundred protein-ligand complexes with known binding affinities, as well as to score the isolated proteins without their bound ligands, based on their respective structure tessellations. Initially, the difference (Δ*Q*) between scores calculated for an entire complex and for its isolated protein is considered as a predictor of binding affinity; however, since these Δ*Q *do not scale as binding free energy values, two hundred randomly selected protein-ligand complexes from this set are used to empirically derive a linear function of Δ*Q *as a model for calculating the binding energy. For this training set, we observe a correlation of *r *= 0.79 between calculated and experimental binding energies, with a standard error of *SE *= 1.98 kcal/mol and a regression line of *y *= 1.18*x*. Validation of this model with the remaining one hundred complexes that were held out yields performance measures of *r *= 0.79 and *SE *= 1.93 kcal/mol. In an application of the method, our model is then used to predict binding energies for an independent and diverse test set of three hundred enzyme-inhibitor complexes, producing results that are consistent with those based on the training and validation data. Finally, we utilize a diverse test set of one hundred protein-ligand complexes to benchmark the binding energy predictions made with our model, and our correlation between calculated and experimental binding energies for this dataset surpasses those of all 15 related methods to which it is compared. A key advantage with our approach is the ability to generate rapid predictions, typically under one second per complex.

## Competing interests

The author declares that they have no competing interests.

## Authors' contributions

MM conceived of the study, implemented the methods, analyzed the data, and wrote the manuscript.

## Supplementary Material

Additional file 1 PDB accession codes for the 1417 macromolecular structures used to derive the atomic four-body statistical potential. http://proteins.gmu.edu/automute/Additional file 1.txt Click here for file

Additional file 2 Three hundred protein-ligand complexes used to train and validate the model. http://proteins.gmu.edu/automute/Additional file 2.txt Click here for file

Additional file 3 Independent set of three hundred enzyme-inhibitor complexes used to test the model. http://proteins.gmu.edu/automute/Additional file 3.txt Click here for file

## Declarations

Publication of this article was funded in part by the George Mason University Libraries Open Access Publishing Fund.

This article has been published as part of *BMC Structural Biology *Volume 13 Supplement 1, 2013: Selected articles from the Computational Structural Bioinformatics Workshop 2012. The full contents of the supplement are available online at http://www.biomedcentral.com/bmcstructbiol/supplements/13/S1.

## References

[B1] Gilson MK Zhou HX Calculation of protein-ligand binding affinities Annu Rev Biophys Biomol Struct 2007 13 21 42 10.1146/annurev.biophys.36.040306.132550 17201676

[B2] Wang R Lai L Wang S Further development and validation of empirical scoring functions for structure-based binding affinity prediction J Comput Aided Mol Des 2002 13 1 11 26 10.1023/A:1016357811882 12197663

[B3] Krammer A Kirchhoff PD Jiang X Venkatachalam CM Waldman M LigScore: a novel scoring function for predicting binding affinities J Mol Graph Model 2005 13 5 395 407 10.1016/j.jmgm.2004.11.007 15781182

[B4] Gohlke H Hendlich M Klebe G Knowledge-based scoring function to predict protein-ligand interactions J Mol Biol 2000 13 2 337 356 10.1006/jmbi.1999.3371 10623530

[B5] Sotriffer CA Sanschagrin P Matter H Klebe G SFCscore: scoring functions for affinity prediction of protein-ligand complexes Proteins 2008 13 2 395 419 10.1002/prot.22058 18442132

[B6] Huey R Morris GM Olson AJ Goodsell DS A semiempirical free energy force field with charge-based desolvation J Comput Chem 2007 13 6 1145 1152 10.1002/jcc.20634 17274016

[B7] Huang SY Zou X An iterative knowledge-based scoring function to predict protein-ligand interactions: I. Derivation of interaction potentials J Comput Chem 2006 13 15 1866 1875 10.1002/jcc.20504 16983673

[B8] Huang SY Zou X An iterative knowledge-based scoring function to predict protein-ligand interactions: II. Validation of the scoring function J Comput Chem 2006 13 15 1876 1882 10.1002/jcc.20505 16983671

[B9] Tang YT Marshall GR PHOENIX: a scoring function for affinity prediction derived using high-resolution crystal structures and calorimetry measurements J Chem Inf Model 2011 13 2 214 228 10.1021/ci100257s 21214225 PMC3046228

[B10] de Berg M Cheong O van Kreveld M Overmars M Computational Geometry: Algorithms and Applications 2008 Berlin, Springer-Verlag

[B11] Summa CM Levitt M Degrado WF An atomic environment potential for use in protein structure prediction J Mol Biol 2005 13 4 986 1001 10.1016/j.jmb.2005.07.054 16126228

[B12] Fogolari F Pieri L Dovier A Bortolussi L Giugliarelli G Corazza A Esposito G Viglino P Scoring predictive models using a reduced representation of proteins: model and energy definition BMC Struct Biol 2007 13 15 10.1186/1472-6807-7-15 17378941 PMC1854906

[B13] Masso M Knowledge-based scoring function derived from atomic tessellation of macromolecular structures for prediction of protein-ligand binding affinity Bioinformatics and Biomedicine Workshops (BIBMW), 2012 IEEE International Conference on: 4-7 October 2012 2012 17 21 10.1109/BIBMW.2012.6470315

[B14] Wang G Dunbrack RL Jr PISCES: a protein sequence culling server Bioinformatics 2003 13 12 1589 1591 10.1093/bioinformatics/btg224 12912846

[B15] Berman H Henrick K Nakamura H Markley JL The worldwide Protein Data Bank (wwPDB): ensuring a single, uniform archive of PDB data Nucleic Acids Res 2007 13 Database D301 303 10.1093/nar/gkl971 17142228 PMC1669775

[B16] Hu L Benson ML Smith RD Lerner MG Carlson HA Binding MOAD (Mother Of All Databases) Proteins 2005 13 3 333 340 10.1002/prot.20512 15971202

[B17] Benson ML Smith RD Khazanov NA Dimcheff B Beaver J Dresslar P Nerothin J Carlson HA Binding MOAD, a high-quality protein-ligand database Nucleic Acids Res 2008 13 Database D674 678 18055497 10.1093/nar/gkm911 PMC2238910

[B18] Barber CB Dobkin DP Huhdanpaa HT The quickhull algorithm for convex hulls ACM Trans Math Software 1996 13 469 483 10.1145/235815.235821

[B19] Pettersen EF Goddard TD Huang CC Couch GS Greenblatt DM Meng EC Ferrin TE UCSF Chimera--a visualization system for exploratory research and analysis J Comput Chem 2004 13 13 1605 1612 10.1002/jcc.20084 15264254

[B20] Mitchell JBO Laskowski RA Alex A Thornton JM BLEEP-Potential of mean force describing protein-ligand interactions: I. Generating potential J Comput Chem 1999 13 1165 1176 10.1002/(SICI)1096-987X(199908)20:11<1165::AID-JCC7>3.0.CO;2-A

[B21] Masso M Vaisman II Accurate prediction of enzyme mutant activity based on a multibody statistical potential Bioinformatics 2007 13 3155 3161 10.1093/bioinformatics/btm509 17977887

[B22] Masso M Vaisman II AUTO-MUTE: web-based tools for predicting stability changes in proteins due to single amino acid replacements Protein Eng Des Sel 2010 13 8 683 687 10.1093/protein/gzq042 20573719

[B23] Sippl MJ Boltzmann's principle, knowledge-based mean fields and protein folding. An approach to the computational determination of protein structures Journal of Computer-Aided Molecular Design 1993 13 4 473 501 10.1007/BF02337562 8229096

[B24] Wang R Lu Y Wang S Comparative evaluation of 11 scoring functions for molecular docking J Med Chem 2003 13 12 2287 2303 10.1021/jm0203783 12773034

[B25] Zhang C Liu S Zhu Q Zhou Y A knowledge-based energy function for protein-ligand, protein-protein, and protein-DNA complexes J Med Chem 2005 13 7 2325 2335 10.1021/jm049314d 15801826

[B26] Velec HF Gohlke H Klebe G DrugScore(CSD)-knowledge-based scoring function derived from small molecule crystal data with superior recognition rate of near-native ligand poses and better affinity prediction J Med Chem 2005 13 20 6296 6303 10.1021/jm050436v 16190756

[B27] Gehlhaar DK Verkhivker GM Rejto PA Sherman CJ Fogel DB Fogel LJ Freer ST Molecular recognition of the inhibitor AG-1343 by HIV-1 protease: conformationally flexible docking by evolutionary programming Chem Biol 1995 13 5 317 324 10.1016/1074-5521(95)90050-0 9383433

[B28] Gehlhaar DK Bouzida D Rejto PA Parrill L, Reddy MR Reduced dimensionality in ligand-protein structure prediction: covalent inhibitors of serine proteases and design of site-directed combinatorial libraries Rational Drug Design: Novel Methodology and Practical Applications 1999 Washington, DC: American Chemical Society 292 311

[B29] Jones G Willett P Glen RC Leach AR Taylor R Development and validation of a genetic algorithm for flexible docking J Mol Biol 1997 13 3 727 748 10.1006/jmbi.1996.0897 9126849

[B30] Meng EC Shoichet BK Kuntz ID Automated docking with grid-based energy evaluation J Comput Chem 1992 13 4 505 524 10.1002/jcc.540130412

[B31] Eldridge MD Murray CW Auton TR Paolini GV Mee RP Empirical scoring functions: I. The development of a fast empirical scoring function to estimate the binding affinity of ligands in receptor complexes J Comput Aided Mol Des 1997 13 5 425 445 10.1023/A:1007996124545 9385547

[B32] Muegge I Martin YC A general and fast scoring function for protein-ligand interactions: a simplified potential approach J Med Chem 1999 13 5 791 804 10.1021/jm980536j 10072678

[B33] Bohm HJ The development of a simple empirical scoring function to estimate the binding constant for a protein-ligand complex of known three-dimensional structure J Comput Aided Mol Des 1994 13 3 243 256 10.1007/BF00126743 7964925

[B34] Bohm HJ Prediction of binding constants of protein ligands: a fast method for the prioritization of hits obtained from de novo design or 3D database search programs J Comput Aided Mol Des 1998 13 4 309 323 10.1023/A:1007999920146 9777490

[B35] Cerius2 version 4.6 http://www.accelrys.com

[B36] Rarey M Kramer B Lengauer T Klebe G A fast flexible docking method using an incremental construction algorithm J Mol Biol 1996 13 3 470 489 10.1006/jmbi.1996.0477 8780787

[B37] Morris GM Goodsell DS Halliday RS Huey R Hart WE Belew RK Olson AJ Automated docking using a lamarckian genetic algorithm and an empirical binding free energy function J Comput Chem 1998 13 14 1639 1662 10.1002/(SICI)1096-987X(19981115)19:14<1639::AID-JCC10>3.0.CO;2-B

